# A nationwide registry for recurrent urolithiasis in the upper urinary tract – The RECUR study protocol

**DOI:** 10.1186/s12913-022-08375-7

**Published:** 2022-08-19

**Authors:** Martin Schoenthaler, Urs Alexander Fichtner, Martin Boeker, Daniela Zoeller, Harald Binder, Hans-Ulrich Prokosch, Friederike Praus, Tabea Walther, Maximilian Glienke, Petar Horki, Christian Gratzke, Erik Farin-Glattacker

**Affiliations:** 1 Department of Urology, Faculty of Medicine, Medical Centre - University of Freiburg, Hugstetter Str. 55, 79106 Freiburg, Germany; 2 Medical Centre, Faculty of Medicine, Institute of Medical Biometry and Statistics, University of Freiburg, Section of Health Care Research and Rehabilitation Research, Hugstetter Straße 49, 79106 Freiburg, Germany; 3 Medical Centre Rechts Der Isar, Faculty of Medicine, Institute of Artificial Intelligence and Medical Informatics, Ismaninger Straße 22, Technical University of Munich, 81675 Munich, Germany; 4 Medical Centre, Faculty of Medicine, Institute of Medical Biometry and Statistics, Division Medical Data Science, University of Freiburg, Zinkmattenstraße 6A, 79108 Freiburg, Germany; 5 Medical Centre, Faculty of Medicine, Institute of Medical Biometry and Statistics, University of Freiburg, Stefan-Meier-Straße 49, 79104 Freiburg, Germany; 6 Institute for Medical Informatics, Biometry and Epidemiology, Wetterkreuz 13, 91058 Erlangen-Tennenlohe, Germany

**Keywords:** Urolithiasis, Recurrent urinary stone disease, Observational study, Registry, Medical informatics, Data integration, Real world data, Risk scores, Quality of life, Treatment evaluation

## Abstract

**Background:**

Urinary stone disease is a widespread disease with tremendous impact on those affected and on societies around the globe. Nevertheless, clinical and health care research in this area seem to lag far behind cardiovascular diseases or cancer. This may be due to the lack of an immediate deadly threat from the disease and therefore less public and professional interest. However, the patients suffer from recurring, sometimes intense pain and often must be treated in hospital. Long-term morbidity includes doubled rates of chronic kidney disease and arterial hypertension after at least one stone-related event. Observational studies, more specifically, registries and other electronic data sets have been proposed as a means of filling critical gaps in evidence. We propose a nationwide digital and fully automated registry as part of the German Ministry for Education and Research (BMBF) call for the "establishment of model registries”.

**Methods:**

RECUR builds on the technical infrastructure of Germany’s Medical Informatics Initiative. Local data integration centres (DIC) of participating medical universities will collect pseudonymized and harmonized data from respective hospital information systems. In addition to their clinical data, participants will provide patient reported outcomes using a mobile patient app. Scientific data exploration includes queries and analysis of federated data from DICs of eleven participating sites. All primary patient data will remain at the participating sites at all times. With comprehensive data from this longitudinal registry, we will be able to describe the disease burden, to determine and validate risk factors, and to evaluate treatments. Implementation and operation of the RECUR registry will be funded by the BMBF for five years. Subsequently, the registry is to be continued by the German Society of Urology without significant costs for study personnel.

**Discussion:**

The proposed registry will substantially improve the structural and procedural framework for patients with recurrent urolithiasis. This includes advanced diagnostic algorithms and treatment pathways. The registry will help us identify those patients who will most benefit from specific interventions to prevent recurrences. The RECUR study protocol and the registry’s technical architecture including full digitalization and automation of almost all registry-associated proceedings can be transferred to future registries.

**Trial registration:**

This study is registered at the German Clinical Trial Register (*Deutsches Register Klinischer Studien*), DRKS-ID DRKS00026923, date of registration January, 11^th^ 2022.

## Background

Urinary stone disease (syn. *urolithiasis*) is a widespread disease with tremendous impact on those affected and on societies around the globe. Nevertheless, clinical and health care research in this area seem to lag far behind cardiovascular diseases or cancer. This may be due to the lack of an immediate deadly threat from the disease and therefore less public and professional interest. There is a paucity of data on epidemiology and health care concerning urolithiasis in Germany and around the world. In Germany, Hesse et al. published the only available data on the incidence and prevalence of urinary stone disease in 2003 [1]. According to their data, the incidence and prevalence of urolithiasis are rising (incidence in 1979: 0.54%, 2001: 1.47%; prevalence 2001: 4.77%). This includes recurrent urolithiasis in 50% of patients within 5 years [2].

### Disease burden and socio-economic impact

The high recurrence rate of urolithiasis is in line with repeated episodes of severe pain and hospitalization. Affected patients may need multiple interventions and operations, including treatment-associated complications. Long-term morbidity includes septicemia, doubled risk of acute or permanent kidney damage, and arterial hypertension after at least one stone-related event, leading to significant limitations in quality of life [3].

In Germany, the numbers of in-hospital treatments for urolithiasis exceed those of any other urological disease including prostate cancer, bladder cancer, and benign prostatic disease [4]. Overall, urolithiasis affects approximately 10% of the population of industrial countries, which corresponds to 8 million patients in Germany [5]. Costs for work days lost and treatment are high exceeding € 500 million and 5.8 million lost working days per year in Germany in 1997 [6]. In the United States, costs of stone disease are projected to exceed $ 4.5 billion annually by 2030 [7].

### Risk factors

To date, little is known about sociodemographic and behavioral risk factors for recurrent urolithiasis [8]. Risk factors explaining recurrent stone formation in the vast majority of recurrent stone formers thus remain unaccounted for. However, evidence is pointing to specific characteristics that correlate with higher risks of recurrent stone forming: Men are more often affected than women (prevalence 5.5% vs. 4.0%) [1]. Fifty percent of patients are considered “high risk” patients with more than two stone-related events and/ or known risk factors for disease recurrence [9]. Risk factors for highly recurrent stone disease include general factors (early onset, family history of urolithiasis), specific stone compositions, diseases associated with stone formation (hyperparathyroidism, gastrointestinal disease, etc.), genetically determined diseases associated with stone formation (cystinuria, primary hyperoxaluria, etc.), and anatomical abnormalities of the urinary tract associated with stone formation [10]. Metabolic evaluations of recurrent stone formers may reveal abnormalities in urine chemistry associated with stone formation [2]. In addition, recent studies identified several gene mutations associated with nephrolithiasis [10].

### Current treatments

Available treatment modalities include medical treatment for renal colic and metaphylaxis of urolithiasis (prevention of recurrence), use of a ureteric catheter to treat hydronephrosis due to ureteral stones, and various surgical treatments to remove urinary stones (shock wave lithotripsy, ureteroscopy and percutaneous nephrolithotomy). Approximately 750.000 stone-related events are treated annually in Germany, with some patients receiving multiple treatments [11]. This includes > 130.000 surgical treatments to remove stones [12]. In the United States, costs for urolithiasis treatments exceed those for prostate cancer and benign prostatic hyperplasia [13].

### Research on urolithiasis

Overall, the level of evidence in urolithiasis-related research is low. In 2014, we reviewed the current literature on clinical urolithiasis-related trials to answer fundamental questions—e.g., “which is the best surgical treatment for patients with upper tract urinary stones?” or “how should we manage patients with recurrent urolithiasis?”.

We found levels of evidence 1 (randomized controlled trials, RCT), 2 (cohort studies), 3 (case–control studies) and 4 (case series) in 15%, 14%, 21% and 51% of trials published in 2014, respectively [14]. The reasons for low numbers of high level trials are:

- Patients refrain from being randomized, especially when established surgical treatment is involved, e.g., shock-wave therapy vs. ureteroscopy.
- Explanatory trials - e.g., RCTs performed with relatively small samples at sites with experienced investigators and highly selected patients, may overestimate benefits and underestimate harm. Results from these trials may not inform practice because they are optimized to determine efficacy (performance of an intervention under ideal and controlled circumstances) as opposed to effectiveness [15].

Observational studies and more specifically registries and other electronic data sets have been proposed as a means of filling critical gaps in evidence [16]. Clinical registries record real-world data to evaluate associations among variables on the individual (e.g. patient demographics, physical and physiologic parameters), the context (e.g. climate, working conditions) and clinical outcomes (e.g. stone recurrence). Information from registries may also be used to evaluate how patients with different characteristics respond to various treatments and to compare the performance of healthcare providers with regard to their outcomes and resource use (*effectiveness*—i.e., performance in ‘real world’ conditions) [17]. Sources of real-world data may include electronic health records and mobile applications.

### Registries in urolithiasis

In 2009 and 2010, the Clinical Research Office of the Endourological Society (CROES) implemented two clinical registries on PNL and URS treatment of urinary stones. Both registries collected data on active treatment for urolithiasis from dedicated centres around the world over a one-year period. Both registries are closed. Data from these registries are limited to the patients' perioperative course. To our knowledge, there have been no nationwide registries on urolithiasis established in Germany or any other industrialized country. However, implementation of a national registry in the U.S. was described in a recent publication [18].

### Objectives

We will implement and assess the effectiveness of the RECUR registry for recurrent urolithiasis in the upper urinary tract as a distributed multi-institutional digital registry based on the technical infrastructure of the German Medical Informatics Initiative (MI-I).

Our purpose is to answer the following research questions:

1. Description of disease burden: What impact does urolithiasis have on individual patients and the health care system?
  Impact of the disease on patient-reported outcomes (PROMs, e.g. working ability, perceived restrictions of activity and participation due to urolithiasis, stone-related quality of life) [19, 20]: trajectories of these outcomes will be analysed
  Socio-economic impact (e.g., length of hospital stay, lost working days) [6, 21]: standardized unit costs will be applied to quantify the costs of medical resource utilization; analyses will be carried out from the healthcare perspective
  Gender-dependent impact of urolithiasis: subgroup analyses will reveal gender-specific aspects of the disease burden [22, 23]
2. Determination and validation of risk factors: How can patients at risk of stone recurrence be identified beyond the known risk factors?
  Personalized medicine: taking into account individual variability in lifestyle and nutritional habits, sociodemographic factors (age, education, socio-economic status), genes [24–26]: multi-level models for change will be devised
  Development of a risk score to predict the probability of stone recurrence
  Validation of the risk score using longitudinal registry data
  Deduction of implications for prevention and treatment
3. Treatment evaluation: Which is the optimum treatment for individual patients? [27]
  Risk-adjusted comparison of outcomes for different treatment options (e.g. surveillance vs. medical treatment vs. surgical treatment): Propensity score matching analyses will be conducted to compare outcomes; this method will help us estimate a given treatment’s effect while accounting for the covariates that predict receiving the treatment.
  We will focus on outcomes relevant to patients such as disease recurrence, sick leave due to urolithiasis, perceived restrictions of activity and participation due to urolithiasis.

## Methods

### Technical infrastructure

RECUR builds on the technical infrastructure of Germany’s MI-I. The BMBF launched its medical informatics funding scheme to make data from healthcare and research more useful and meaningful [28]. The BMBF provides funding for several consortia within the scope of the MI-I. Each consortium comprises multiple university hospitals and additional partners. Key to the initiative is the establishment and interconnection of DIC by each consortium [28].

The MIRACUM Consortium (Medical Informatics for Research and Care in University Medicine) unites ten university hospitals, two universities, and a partner from the healthcare industry. Its goal is to make clinical, image and molecular/genomic data available for use in innovative research projects – both within and across multiple institutions [29]. The participating sites are the University Hospitals in Dresden, Erlangen, Frankfurt, Freiburg, Giessen, Greifswald, Magdeburg, Mainz, Mannheim, Marburg and Malteser Waldkrankenhaus in Erlangen.

The features of a clinical data warehouse (CDWH) include data integration, consolidation, and presentation [30]. Data integration is the merging of information from heterogeneous data sources, often from primary systems. In this way, the primary systems are uncoupled from further processing of the data to maintain their stability. Consolidation brings heterogeneous information together in a structured data model consisting of facts (e.g., primary and secondary diagnosis recorded for a patient) and dimensions (i.e., international statistical classification of diseases and related health problems (ICD) catalogue). In the subsequent presentation (e.g., reporting and interactive queries), such harmonized content can be used more effectively than would be possible with direct access to the individual sources. Notable examples of software platforms and data models for CDWH are i2b2, TranSMART, and OHDSI (observational medical outcomes partnership/observational health data sciences and informatics) [31]. These platforms have enabled the successful (re)use of electronic health records (EHR) in various fields, for example, disease genomics and pharmacovigilance [32].

All clinical data in RECUR will be retrieved from hospital information systems (HIS) and EHR of individual patients via DICs of the participating centres of the MIRACUM consortium. Data will remain in the local centres (decentralized or distributed registry). In addition, patients will provide patient-reported outcomes using a RECUR smartphone app. These data are also processed and stored by the DICs of the participating sites.

Analyses will be performed by submitting electronic analysis requests to the DICs according to the federated MIRACUM analysis concept.

### Target population and recruitment

The target population includes all patients with recurrent urinary stone disease (approximately 5% of the population of industrialized countries). The source population comprises all patients with recurrent urinary stone disease (≥ 2 stone-related events) evaluated and treated at participating hospitals of the MIRACUM consortium. Observational units are all patients who give their consent to participate.

Eligible patients will be offered a patient information brochure in participating centres during outpatient or inpatient visits. This brochure informs patients about the purpose of the planned registry. At the same time, the personal benefits of participation will be explained and health professionals in recruiting centres will provide detailed information if necessary. No financial incentives will be provided for participation.

If interested in participation, patients can use a downloadable app to consent and register. To increase adherence, patients can contact the study team in case of upcoming study-related questions or the technical support in case of technical problems.

### Sample size calculation

As with many registry studies investigating widespread diseases with high prevalence, sample size is not a major concern. Therefore, we did not perform sample size calculations. We aim at collecting as much and as complete data as possible by reducing a) the effort for including patients into this study and b) the size of the questionnaires to a minimum. Non-responders to the app-integrated questionnaires will automatically receive reminders to increase completeness of data. Considering the high volume of eligible patients at the participating centres, we expect an annual recruitment rate of 500 to 1000 patients.

### The RECUR patient app

Patients will be included in this study based on a signed electronic informed consent. Within this smartphone application, participants will be provided with a comprehensive patient information form. Participants are asked to sign the digital consent and to confirm that they had the opportunity to ask their treating physicians or the study team to answer any open questions concerning the registry and that they have read and understood the patient information.

After electronic consent, the RECUR app will provide participants with the digital questionnaires regarding sociodemographic data, quality of life, working ability, nutrition and physical activity. The data will be collected anonymously without identifying features such as an IP-address or user name. All data is stored securely in a public cloud infrastructure. Participants will have to present in-person to a participating centre in order to establish a pseudonymous link between their in-app responses and their EHR. Subsequently, the participating clinic can merge the patients’ existing and future responses with clinical routine data in its DIC.

Participants will receive digital questionnaires at regular intervals, e.g. monthly, to answer certain questionnaires. If patients do not complete the questionnaires, they will receive a reminder after 4 weeks. We will collect follow-ups at semiannual intervals. PROM results will also be available to both patients and their treating physicians via a local interface between patient app and hospitals using the pseudonymized link as described above. This will enable patients and their doctors to discuss the patient’s personal risk profile based on clinical findings and PROMs.

Participants may discontinue the study at any time using one of the following options: uninstalling the RECUR App, withdrawing their given consent within the app, or clicking the option to delete all personal data. It will not be possible to delete data that has already been processed.

### Data management and data safety

Clinical data for the RECUR distributed registry will be captured, processed, stored and analysed using established MI-I/ MIRACUM tools. All primary data will remain untouched in local HIS. All scientific data used for RECUR will be processed and stored in research data repositories after transfer into each DIC. No central repository will be established. We will use the established MI-I tools for standardization of data, their quality assurance and protection. The data protection policy of RECUR includes the following: (1) pseudonymization of data stored in research data repositories, (2) data security and protection measures on the level of the computational centre of the participating centres, (3) distributed privacy-preserving analysis tools (no transfer of individual patient data between sites or a central server).

Key performance indicators (number of participating patients, frequency of patients providing PROMs, achievement of milestones) and data quality measures (completeness, correctness, data accuracy) of the registry will be checked regularly at each site. A performance and quality report will be generated centrally at the Medical Centre—University of Freiburg at regular intervals.

### Data set

The full data set will be composed out of three sets of characteristics. This includes the participants’ clinical data (diagnoses, treatments, laboratory data, radiology findings, medication) available via the data integration centres (catalogue of characteristics [COC] I). COC II contains all patient reported outcome measures (PROMs) as provided by the participants via the RECUR smartphone application. A third COC will contain all information retrieved by yearly follow-ups of the clinical DIC data and PROMs via the patient app.

### Outcomes

Primary outcome variables of the registry are “stone recurrence” and “stone-related quality of life” using the German version of the Wisconsin Stone Quality of Life Questionnaire (WISQOL) [33]. Secondary outcomes include pain, functional restrictions, working ability, sick leave, and sequelae. Determining and confounding variables include individual and external risk factors.

Individual risk factors include clinical findings (size, weight, co-morbidity), laboratory results (including stone composition), radiology reports (including number, size and location of stones), medication, etc. (captured via DICs) and sociodemographic and lifestyle variables (nutrition, physical activity). Patients will be divided into risk groups to determine the effectiveness of different treatments. External risk factors include all interventions such as secondary prevention measures, non-surgical and surgical treatments for urolithiasis.

### Data analysis

To answer research question 1 (description of disease burden), descriptive analysis methods will be used. The WISQOL will be analysed following the specifications of the developers [33]. No monitoring of PROMs during the study will take place. We will carry out an analysis of patterns of missing items and will take appropriate measures to deal with them.

Regarding research question 2 (determination and validation of risk factors), we expect missing data, that might occur not completely at random. Therefore, multiple imputations will be applied to reduce the chance of bias from missing data. Two-level multiple logistic regression models with the dependent variable stone recurrence will be conducted to account for cluster effects (patients are nested within centres). By using Receiver Operating Characteristics (ROC) analyses, we will determine the predictive power of the risk factors. The Youden Index and area under the curve (c statistic) will be calculated. As ROC curves do not consider time to event and right censoring, we will complement this analysis via survival analysis techniques like Kaplan–Meier or Cox regression analyses.

Since prognostic models derived from multivariable regression analysis lead to overestimated predictions when applied in new patients, we will internally validate our model with bootstrapping techniques. For external validation, it is essential to evaluate the performance of the model in a sample independent from those used to develop the model. This will be realized by comparing the prognostic capacity in the data set acquired in the first year with that in the second year.

For treatment evaluation (research question 3), propensity score matching will be applied. This method is increasingly used as an alternative to traditional regression to balance differences between treatment groups in descriptive and causal comparisons. To account for the nested data structure, propensity-score-weighted estimators for clustered data will be applied. Analyses corresponding to research question 1 will be conducted every six months, the other analyses annually.

### Machine learning

In order to identify patient subgroups and treatment patterns, we will apply machine learning techniques, in particular variational auroencoders (VAEs) as a deep learning technique. This will allow us to cluster patients in an unsupervised manner, also taking time structure into account. This will be complemented by more standard descriptive statistics for comprehensively describing the characteristics of different patient subgroups, the disease course, and treatment paths. In years 1 and 2, this approach will primarily be used for quality control, whereby identified patterns will reveal artefacts in the data that need to be resolved, and help us prioritize measured patient characteristics for quality improvement. In year 5, the techniques will be deployed on a final analysis data set for identifying prognostically relevant patient subgroups, while also guiding propensity score development for the causal analyses described above. All analyses will be pre-specified in a statistical analysis plan.

## Discussion

The RECUR study is part of the German ministry for Education and Research call for the "establishment of model registers”. The registry will help us identify those patients who will most benefit from specific interventions to prevent recurrences. In addition, the registry’s proposed technical architecture built on federated DICs of different hospitals can be transferred to future registries.

The full digitalization and automation of almost all registry-associated proceedings – from patient inclusion to data acquisition and processing – will effect various limitations. The necessary use of new information technologies such as a smartphone may lead to a preferred selection of young, well-educated and German native-speaking patients. We will estimate the extent of bias by comparing basic characteristics of the study population and the overall group of urolithiasis patients at the participating centres. The increasing use of smartphones across all age and sociodemographic groups will reduce this bias in the future.

A considerable proportion of potential participants may have reservations regarding the automated acquisition, storage and use of their data. We will attempt to address these concerns through a transparent and comprehensive information process. This will include both a comprehensive informed consent form and web-based animated illustrations. In addition, patients will be encouraged to ask the medical staff and their treating physicians at any time if needed.

Retention of patients and adherence to the schedule of questionnaires may be another issue of concern. All participants will be asked to answer questionnaires on a regular basis over a long period. This could be particularly true for participants who have not had any stone-related symptoms for a long time. We established a number of strategies to encourage participants to stick to the project. This includes a pleasing design of the RECUR website and patient app with continuous updates and information relevant to patients with recurrent stone disease. Patients can also opt-in to receive a corresponding RECUR newsletter. We will establish a follow-up mechanism in order to get information on the reasons for patient dropouts. Based on the results, our strategies on patient adherence may be adjusted.

The results of our study may not be fully applicable to all stone patients, as almost all participating centres are highly specialized including 10 of 36 German university hospitals. In future, however, further university and non-university hospitals are to be included in the digital network of the MI-I and the RECUR registry.

Most statistical limitations may be reduced by the expected large sample size. Based on an average number of more than 200 eligible patients per centre per year, we expect a recruitment rate of 500—1000 patients per year. Based on the registry’s technical framework, we believe that a very high number of patients will be recruited in the future by including more hospitals into the nationwide digital network of the MI-I.

We believe that the proposed registry will substantially improve the structural and procedural framework for patients with recurrent urolithiasis. This includes advanced diagnostic algorithms and treatment pathways. The registry will help us identify those patients who will most benefit from specific interventions to prevent recurrences. The RECUR Study protocol and the registry’s technical architecture including full digitalization and automation of almost all registry-associated proceedings can be transferred to future registries.

## Data Availability

Data sharing is not applicable to this article as no datasets have been generated or analysed yet. De-identified individual clinical trial participant-level data (IPD) will be generated at participating sites of this trial. IPD will not be shared between sites and not with (scientific) public. However, participating centres are asked to formulate scientific questions and to compile the necessary data sets. In addition to RECUR’s general ethics approval, individual studies based on the RECUR registry’s data sets will need approval by the submitting centre’s ethics committee. Furthermore, local use & access committees of all centres will review queries of a specific centre’s study group and agree to provide aggregated data, if applicable. Along with this manuscript, we provide the SPIRIT Checklist to improve the completeness of this study protocol. Since patient-reported outcomes are measured within this study, we followed the enhanced SPIRIT-PRO Checklist including a guideline how to handle PROs in clinical trial protocols [34].

## Abbreviations

RECUR: A nationwide registry for recurrent urolithiasis in the upper urinary tract
BMBF: German Ministry for Education and Research
DIC: Data integration centre
DRKS: Deutsches Register Klinischer Studien
RCT: Randomized controlled trial
CROES: Clinical Research Office of the Endourological Society
PNL: Percutaneous nephrolitholapaxy
URS: Rigid and flexible ureterorenoscopy
MI-I: Medical Informatics Initiative
MIRACUM: Medical Informatics for Research and Care in University Medicine
CDWH: Clinical data warehouse
ICD: International statistical classification of diseases and related health problems
HER: Electronic health records
HIS: Hospital information system
PROMS: Patient-reported outcome measure
COC: Catalogue of characteristics
WISQOL: Wisconsin Quality of Life Questionnaire
ROC: Receiver Operating Characteristics
IPD: Individual clinical trial participant-level data
